# Applying Behavioural Insights to HIV Prevention and Management: a Scoping Review

**DOI:** 10.1007/s11904-022-00615-z

**Published:** 2022-08-05

**Authors:** Alexsandra Andrawis, James Tapa, Ivo Vlaev, Daniel Read, Kelly Ann Schmidtke, Eric P. F. Chow, David Lee, Christopher K. Fairley, Jason J. Ong

**Affiliations:** 1 Melbourne School of Population and Global Health, University of Melbourne, Melbourne, Australia; 2 Central Clinical School, Monash University, Melbourne, Australia; 3 Warwick Business School, Coventry, UK; 4 Warwick Medical School, Coventry, UK; 5 Melbourne Sexual Health Centre, Melbourne, Australia; 6 Faculty of Infectious and Tropical Diseases, London School of Hygiene and Tropical Medicine, London, UK; 7 Carlton, Australia

**Keywords:** HIV, Behavioural economics, Nudge, Behavioural insights

## Abstract

**Purpose of Review:**

This scoping review summarises the literature on HIV prevention and management interventions utilizing behavioural economic principles encapsulated in the MINDSPACE framework.

**Recent Findings:**

MINDSPACE is an acronym developed by the UK’s behavioural insights team to summarise nine key influences on human behaviour: Messenger, Incentives, Norms, Default, Salience, Priming, Affect, Commitment, and Ego. These effects have been used in various settings to design interventions that encourage positive behaviours. Currently, over 200 institutionalised behavioural insight teams exist internationally, which may draw upon the MINDSPACE framework to inform policy and improve public services. To date, it is not clear how behavioural insights have been applied to HIV prevention and management interventions.

**Summary:**

After screening 899 studies for eligibility, 124 were included in the final review. We identified examples of interventions that utilised all the MINDSPACE effects in a variety of settings and among various populations. Studies from high-income countries were most common (*n* = 54) and incentives were the most frequently applied effect (*n* = 100). The MINDSPACE framework is a useful tool to consider how behavioural science principles can be applied in future HIV prevention and management interventions. Creating nudges to enhance the design of HIV prevention and management interventions can help people make better choices as we strive to end the HIV/AIDS pandemic by 2030.

**Supplementary Information:**

The online version contains supplementary material available at 10.1007/s11904-022-00615-z.

## Introduction

HIV continues to be a major public health issue. In 2020, 37.7 million people were living with HIV, with 1.5 million new infections globally [1]. Multiple HIV prevention and management interventions are needed to achieve the Joint United Nations Programme on HIV/AIDS (UNAIDS)’s 95–95-95 targets by 2030. Biomedical prevention interventions, including pre-exposure prophylaxis (PrEP), provide an effective HIV prevention strategy, and the use of antiretroviral therapy (ART) for HIV management closes the gap on life expectancy outcomes between affected patients and the general population [2]. However, HIV prevention and management strategies are often underutilised in key populations such as men who have sex with men (MSM), intravenous drug users, and sex workers [3–5].

HIV prevention and management interventions grounded in psychological theories could make them more effective in that these understandings of human behaviours underpin the design and implementation of health interventions. Behavioural economics blends psychological and economic principles to provide unique insights into decision-making and behaviour change. Behavioural economics posits that human decision making is influenced by heuristics and cognitive biases. People adapt decision-making depending on resource constraints, context, and other exogenous factors, including whether decisions are made using automatic (system 1 — inherent biases and heuristics) or deliberative processing (system 2 — rational, reasoned thinking) [6].

Nudging is one popular application of the principles described in behavioural economics. A nudge is defined as any aspect of the choice architecture that predictably alters behaviour without forbidding any options or significantly changing their economic incentives [7]. Nudges tend to be low cost and preserve the freedom to choose, and can be used to overcome suboptimal behaviour [8]. Examples of nudges include automatic enrollment into employee retirement savings [9], attaching a fly-shaped sticker on urinals to reduce spillage [10] and highlighting the positive behaviour of others, for instance ‘9 out of 10 people pay their tax on time’ [11]. Nudges used in public health interventions to encourage behaviour change can be summarised in the behavioural economics framework ‘MINDSPACE’ [12]. MINDSPACE is an acronym that summarises nine effects on people’s behaviour that, once realised, can be harnessed to influence their future decisions and ultimately improve public health (Box 1).

We used a scoping review methodology to assess the extent of HIV prevention and management interventions which can be described by behavioural economic principles, and highlight gaps whereby use of behavioural economic principles may further enhance the design of future interventions.

**Box** **1** Definitions of the MINDSPACE effects [12]

**Table:** 

	Definitions
Messenger	Who communicates the information
Incentive	Response to incentives can depend on how they are presented
Norms	People are influenced by what others do
Default	Preset options will be activated unless an active choice occurs
Salience	Attention is drawn to something novel or that seems relevant to the target population
Priming	Exposure to subconscious cues may influence people’s performance or choice on a subsequent task
Affect	Emotional associations that shape actions
Commitment	Consistency with public promises and reciprocate acts
Ego	Acting in ways to make one feel better about themselves

## Methods

We used Arksey and O’Malley’s scoping review methodology to examine the literature on HIV prevention and management interventions which can be described by behavioural economics principles [13]. This consisted of five stages: (1) identification of a research question; (2) identification of relevant articles; (3) selection of articles; (4) extraction and charting of data; and (5) synthesizing, summarizing, and reporting the results.

### Search Strategy

Our search included studies published between January 2000 and August 2021 on HIV prevention and management interventions using behavioural economic principles. We searched the following databases on 11th August, 2021: Medline, PsycInfo, Scopus, and CINAHL. We used the following search terms: HIV, AIDS, prevention, testing, medication, PrEP, PEP, condoms, incentives, reinforcement, economic, and behavioural economics. Detailed information of search terms used in the search strategy is listed in the Appendix (Supplementary Table 1).

### Inclusion and Exclusion Criteria

Studies were included if they contained information to describe HIV prevention interventions (such as HIV testing, PrEP) or HIV management interventions (such as linkage to care, ART medication adherence). We excluded studies not published in English, only described theoretical principles, and systematic or literature reviews.

### Screening and Data Extraction

After removing duplicate studies, the titles and abstracts of the remaining articles were independently evaluated for relevance by two reviewers (AA and JT) using Covidence systematic review software (Veritas Health Innovation, Melbourne, Australia). Any discrepancies were assessed by a third reviewer (JO). Similarly, data were independently extracted by two reviewers (AA and JT) with any discrepancies assessed by a third reviewer (JO). The following data were extracted: authors’ name, year of publication, paper title, country and purpose of the intervention, intervention description, study period, recruitment site, and demographic characteristics of targeted populations.

### Data Analysis

We report our findings using the PRISMA for scoping review guidelines [14]. We categorised the interventions according to their deployment of the nine MINDSPACE categories. The studies were further grouped based on whether the intervention applied to HIV prevention or management. We also grouped interventions by country income level according to the World Bank classification [15]. We used descriptive statistics to summarise the characteristics of the included studies. While we cannot give an account of all the studies, we describe a few key studies in the text, illustrating the wide range of ways the nudge effects (using the MINDSPACE framework) may describe the interventions deployed in the field. We provide the list of relevant studies for each effect in Table 2.

## Results

**Fig. 1:**
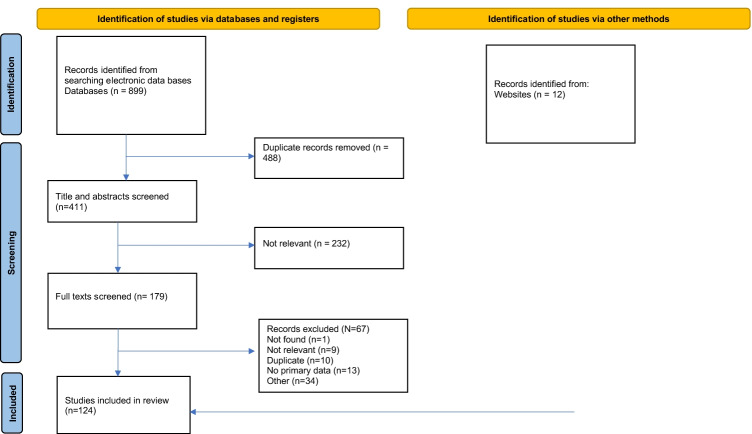
PRISMA flowchart. This figure has been created by the authors for the purposes of this research. No permission is needed

**Fig. 2:**
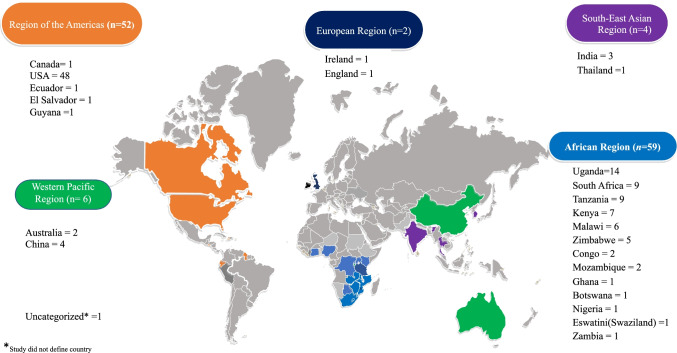
Studies categorised by countries in WHO regions (*N* = 124). This figure has been created by the authors for the purposes of this research. No permission is needed

Figure 1 depicts the study screening process and article selection as a PRISMA flowchart. We found 899 studies and included 124 in this scoping review. The characteristics of the studies are summarised in Table 1. In total, 63 studies were related to HIV prevention, and 63 were related to HIV management (with two studies related to both prevention and management). Most were from high-income countries (*n* = 54) and most HIV prevention (*n* = 36) and management (*n* = 41) interventions targeted the general population. Figure 2 categorises the countries according to the World Health Organization (WHO) regions, with most studies taking place in Africa (*n* = 60) and the Americas (*n* = 52).

**Table 1:** Characteristics of studies included in the scoping review

	*n*	%
Country income level (*N* = 124)		
High	54	43.5
Middle	47	37.9
Low	21	16.9
Populations targeted in HIV prevention studies (*N* = 63)		
Female sex workers	2	3.2
Men who have sex with men	12	19.0
Transgender women*	4	6.3
Substance users	5	7.9
Children and young adults**	10	15.9
Mothers/pregnant women	5	7.9
General population	36	57.1
Populations targeted in HIV management studies (*N* = 63)		
Female sex workers	1	1.6
Men who have sex with men	4	6.3
Transgender women*	1	1.6
Substance users	8	12.7
Children and young adults**	9	14.3
Mothers/pregnant women	2	3.2
General population	41	65.1
MINDSPACE		
Messenger	59	47.6
Incentive	100	80.6
Norms	17	13.7
Default	10	8.1
Salience	48	38.7
Priming	13	10.5
Affect	14	11.3
Commitment	50	40.3
Ego	34	27.4

^*^Studies in this category may also include MSM as they did not distinguish TGW and MSM

^**^Studies in this category aimed to recruit 10–25-year-old

Note: Some studies consisted of more than one category so the numbers may not align with total

### MINDSPACE Effects

Incentives were the most popular application of MINDSPACE (*n* = 100) followed by messenger (*n* = 59). A complete list of included HIV prevention and management studies can be found in Supplementary Tables 2 and 3. The summary of included studies categorised as each MINDSPACE effect can be found in Table 2, split by those aimed at HIV prevention and those aimed at HIV management. The studies utilizing each effect are now summarised.

**Table 2:** Summary of the use of MINDSPACE for HIV prevention and management studies

	Examples	References
Messenger		
HIV prevention	Peer groups and/or mentoring in HIV prevention methods	[16–28]
	Clinical staff, case managers, and community HIV programmes to facilitate HIV prevention	[28–40]
	Respected medical establishment to facilitate HIV prevention	[41–48]
HIV management	Case managers to facilitate recruitment into the program and HIV care	[49–56]
	Hospitals and well known/respected sites to improve adherence to treatment and care	[48, 57–74]
Incentives		
HIV prevention	Lottery-based incentives for HIV prevention	[24, 25, 28–30, 33, 39, 75–78]
	Loss framing to promote HIV testing	[79–81]
	Matched savings accounts, deposit contracts, or micro-enterprise tools used to minimise HIV risk behaviours	[27, 31, 82–84]
	Incentives to reinforce HIV prevention education	[5, 27, 85–88]
	Financial incentives for peer and community-based recruiters to encourage HIV testing or VMMC	[16, 19, 23, 26, 36]
	Cash payment to encourage HIV testing	[22, 34, 37, 88–94]
	Financial incentive to take PrEP	[95]
	Cash payment to remain HIV negative	[96, 97]
	Travel costs reimbursement to engage in HIV prevention	[21, 88, 98]
	Non-cash incentives such as personal hygiene products, t-shirts, smartphone credits, gift cards, or food to engage in HIV prevention	[20, 35, 99–102]
HIV management	Financial incentives to reduce HIV viral load or maintain viral load suppression	[38, 49, 55, 58, 62, 69, 103–113]
	Lottery-based incentives contingent on maintaining viral suppression	[52, 53, 56, 59, 73, 114, 115]
	Loss framed whereby incentive was reset if viral load goals were not met	[57]
	Incentives to attend routine HIV management appointments	[51, 54, 63, 64, 116–119]
	Financial incentive for initiating ART/improving adherence	[48, 68, 72]
	Savings account for ART adherence	[66]
	Financial incentive contingent on non-reactive stimulant sample	[120]
	Non-financial incentives: Mobile phone, data or minutes and other electronic devices for ART initiation, reduction of viral load, linkage, and retention in HIV care Food vouchers to improve adherence to treatment and retention in HIV care Culturally meaningful pillboxes to improve adherence to antiretroviral therapy and retention in care	[70, 121–125] [60, 71, 126, 127] [128]
Norms		
HIV prevention	Gender norms to encourage HIV testing and facilitate education	[5, 99, 101]
	Social norms related to HIV prevention, including HIV testing and learning HIV results	[16, 22, 26, 28, 129–131]
	Social norms and gender norms to describe transactional sexual relationships and minimise HIV risk	[31]
	Subjective norms and workplace HIV counselling and testing	[132]
HIV management	Social norms to improve adherence to antiretroviral therapy and retention in care	[58, 72, 128]
	Gender norms and barriers to HIV management	[67]
	Health seeking norms to improve viral suppression	[53]
Default		
HIV prevention	Opt-out HIV screening	[41, 42, 44–47, 133]
	Opt-in, opt-out, and active choice HIV screening	[43, 93]
	Cost-effectiveness of opt-in/out from a hospital perspective	[94]
Salience		
HIV prevention	Text-based messaging to facilitate HIV prevention and linkage to care	[28, 35, 82, 88, 95, 134]
	Incentive-based HIV prevention and retention in care	[22, 31, 83, 111, 135]
	Respected messenger to increase engagement in HIV prevention, with/out financial incentives	[17, 21, 24, 25, 36, 100, 136]
	Culturally relevant and gender-specific messaging to engage in HIV prevention	[30, 101]
	Loss framed lottery intervention to encourage dual contraception methods to prevent HIV infection	[86]
HIV management	Text messages to support linkage to care/medication adherence	[72, 116, 121, 126]
	Lotteries and motivational SMS messages to encourage ART linkage/adherence/viral suppression	[59, 69, 119, 123, 124]
	Computer-based HIV education/motivational modules or mindfulness exercises to reduce HIV viral load	[50, 137, 138]
	Salience increased via incentives for ART initiation, viral suppression, or retention to services	[38, 48, 51, 53, 54, 57, 103, 106, 117, 135]
	Appointment/ART medication reminders	[70, 74, 105, 107, 128]
Priming		
HIV prevention	Use of images of peers in campaigns to encourage HIV testing	[139]
	Contact details of local VCT clinics in banner ads on gay websites	[140]
	Use of prompt to encourage attending HIV testing clinic	[141]
	Daily phone-based PrEP and HIV education modules as well text reminders to take PrEP medication	[142]
HIV management	Use of priming stimuli that is empowering or culturally meaningful to improve adherence to ART and retention in care	[143, 144]
	Use of financial rewards as priming to improve adherence to ART and to suppress viral load count	[49, 145]
	Providing safe sex materials as priming to practice safe sex to prevent HIV transmission	[146]
	Personalised cues and reminder messages for remembering dose times to support ART adherence	[72, 74, 147, 148]
Affect		
HIV prevention	Creating positive emotion for HIV testing or accessing HIV services	[111, 139, 149, 150]
	Peer-led group sessions to learn skills for self-efficacy and positive sexual health behaviours	[31]
	Increasing risk perception towards HIV to encourage HIV prevention behaviours	[99, 151]
HIV management	Creating positive emotion for HIV care retention and ART adherence through social, financial, or non-financial support	[99, 111, 143, 146]
	Group sessions targeting positive affect to increase skills for self-efficacy to encourage ART adherence and viral load suppression	[146, 152–154]
	Motivational messages to encourage ART adherence	[147, 150, 155]
Commitment		
HIV prevention	Use of binding contracts with financial deposits to encourage HIV testing or clinic visits	[139, 156–159]
	Use of non-binding/ soft contracts that target the cognitive dissonance aspect to encourage HIV testing and clinic visits	[141, 160]
	Use of non-explicit commitment devices, such as rewards, for meeting a specified HIV prevention health behaviour in the future (e.g. maintaining HIV negative status)	[161–168]
HIV management	Use of non-explicit commitment devices in the form of financial incentives for ART adherence and viral load suppression goal in the future	[108, 144, 147, 154, 169–181]
	Use of non-explicit commitment devices in the form of financial incentives to meet HIV testing, linkage to HIV care and clinic attendance goal in the future	[172, 182, 183]
	Use of non-explicit commitment devices in the form of non-financial incentives for ART adherence and viral load suppression goal in the future	[74, 147, 155]
	Use of non-explicit commitment devices in the form of non-financial incentives to meet HIV testing, linkage to HIV care and clinic attendance goal in the future	[117, 143, 146, 184–186]
Ego		
HIV prevention	HIV education sessions alongside financial education programmes to target ego and self-efficacy	[18, 31, 99, 187–189]
	HIV education sessions and peer support to target ego and increase HIV risk perception	[151, 190]
	Economic intervention to target ego and promote HIV prevention behaviours	[97, 158, 191]
	Peer support and positive messaging to target ego and encourage HIV prevention behaviours	[139, 149]
HIV management	HIV education or personalised HIV support to encourage HIV linkage and care	[98, 99, 137, 184, 189]
	Financial intervention to target ego and encourage viral load suppression	[66, 145, 152, 154, 178–180, 192, 193]
	Positive messaging, reminders, or motivational interviewing to target ego and encourage viral load suppression	[74, 155, 169, 182, 194]

### Messenger

Thirty-one HIV prevention studies drew on the messenger effect, using a wide range of messengers and messages. For example, one multi-level community-based intervention in El Salvador utilised peers as messengers, both as recruiters to encourage hard-to-reach crack users to test for HIV and as counsellors to reduce sexual risk-taking behaviour [16]. A population-level intervention in Malawi used nurses as messengers. They offered HIV tests to participants in their home and simultaneously discussed HIV prevention strategies [29]. Other messengers included respected community HIV programmes involved in the recruitment of study participants (Table 2).

Twenty-six HIV management interventions drew on the messenger effect. This included a prospective RCT among non-adherent people living with HIV in the United States of America (USA). The intervention utilised a respected HIV/AIDS health care provider to provide individualised intensive case management [49]. Another example was a clinical trial to test the effect of a nurse-led motivational group intervention on adherence to ART and risk reduction behaviours among women living with HIV in the USA. The nurse facilitators used motivational interviewing techniques to explore discrepancies between current behaviours and values [50]. Other messengers included hospitals and well-known/respected sites to improve adherence to treatment, and care and case managers to facilitate recruitment into the programme and HIV care (Table 2).

### Incentives

Fifty-one HIV prevention studies utilised incentives. For example, a study in rural Uganda used an RCT to explore the effectiveness of incentive strategies at high and low amounts to promote HIV testing among men. The study utilised loss framing and lotteries to promote HIV testing where participants were informed they had won a prize, asked to select a prize from various items, then told they would lose the prize if they did not obtain an HIV test. Those who got tested received a further opportunity to win larger prizes as part of a lottery [79]. Another example was a USA-based RCT for people who used drugs. The intervention consisted of a computer-based HIV prevention education programme designed to teach participants about PrEP. The intervention consisted of four modules each with 112 questions. Participants could earn $0.02 for each correct answer given [85]. Other incentive types, including lottery-based incentives, matched savings accounts, deposit contracts, or micro-enterprise tools to minimise HIV risk behaviours, remain HIV negative or participate in voluntary medical male circumcision (VMMC) (Table 2).

Forty-seven HIV management studies utilised incentives. This included an RCT in Uganda among men living with HIV. The financial incentives used contingency management principles to motivate participants in the intervention group to be virally suppressed. Specifically, incentives increased if they were virally suppressed but was reset to the initial value of 4 USD if participants were not virally suppressed [57]. An observational cohort study in rural Uganda utilised short message service (SMS) and travel reimbursements to improve HIV care following an abnormal CD4 test result. Participants with an abnormal CD4 test received SMS notification of their results daily, up to a maximum of 7 days. Participants who returned to the clinic within 7 days of the first message received a transportation reimbursement to cover the cost of transportation to the clinic [116]. Other incentives included lottery-based incentives contingent on maintaining viral suppression and non-financial incentives such as a mobile phone, data or minutes for ART initiation, reduction of viral load, linkage to care, and retention in HIV care (Table 2).

### Norms

Twelve HIV prevention studies used gender, social, and subjective norms. This included a cluster RCT in South Africa that implemented a tablet-based app (EPIC-HIV) to provide a male-targeted intervention to support their HIV testing decision-making process. The content used local narrative and provided information about the likely outcomes of testing [99]. Another RCT provided funding for very small-scale businesses coupled with HIV education sessions targeting female sex workers in India. Group norms were developed at the onset via group rules for HIV prevention sessions which were interactive and didactic [5].

Five HIV management studies used gender, social, and health-seeking norms. Social norms were utilised in an intervention in Ghana. Participants received financial incentives based on group average viral loads and group average clinical attendance. The intervention also utilised peer support where group members chose the group name to provide a sense of identity [58]. A quasi-experimental pilot study in Tanzania explored social norms coupled with a positive priming image known to participants of a Baobab tree (the ‘tree of life’) to improve adherence to antiretroviral therapy and retention in care. This intervention utilised culturally relevant imaging paired with a widely known Tanzanian idiom to relay the group was working together to achieve a goal, implying the support available from other patients, staff, and the community [128].

### Defaults

Ten HIV prevention studies used default options. This included an RCT where participants were designated into either an opt-in, active choice or opt-out HIV screening arm in an emergency department (ED) in the USA. The following text was provided: Opt-in, ‘You can let me, your nurse, or your doctor know if you’d like a test today’; active choice, ‘Would you like a test today?’; or opt-out, ‘You will be tested unless you decline’ [195]. A prospective mixed-methods study in Australia examined healthcare providers’ acceptability of opt-out HIV testing and how it impacted HIV testing rates among homeless and marginalised patients [133]. Other studies using defaults in HIV screening can be found in Table 2.

We did not find any HIV management studies which used the default option.

### Salience

Twenty-one HIV prevention studies used salience. This included an RCT for justice-involved MSM and transgender women (TGW) in the USA, which used peer mentor support and a client-driven approach to address and track preventative health care goals via app-based technology with in-built medication and appointment reminders, to encourage PrEP usage and HIV testing [17]. A pilot RCT among Latinx MSM and TGW in the USA used culturally specific text messaging providing sexual health information coupled with frequent feedback to the health information responses to keep participants engaged [30]. Further examples can be found in Table 2.

Twenty-seven HIV management studies used salience. This included a pilot RCT among adults living with HIV with unsuppressed viral loads in the USA. This intervention used daily lotteries tied to the opening of a wirelessly enabled electronic pill bottle. Participants received an additional incentive if they were virally suppressed at three months. Participants received 1 of 4 daily feedback depending on adherence the day before [59]. An RCT in Uganda provided escalating incentives of $4, $8, and $12.50 for being virally suppressed at 6, 12, and 24 weeks. These incentives aimed to offset the costs of retention in HIV care, as well as provide a reward for viral suppression [57]. Other examples include text-based ART medication reminders and mindfulness exercises to reduce HIV viral load (Table 2).

### Priming

Four HIV prevention studies used priming. Priming stimuli included images of overseas-born MSM in posters and videos in a public health campaign in Australia to encourage international MSM students to seek HIV/STI testing at health facilities [139] or displaying contact details for local voluntary counselling and testing (VCT) clinics in banner ads on the front pages of national gay dating websites to encourage the website users to seek HIV testing [140]. Another study used PrEP education and provision of medication adherence feedback and reminders as priming stimuli to improve the rates of PrEP medication adherence among an at-risk young MSM population for HIV prevention [142] (Table 2).

Eleven HIV management studies used priming. An intervention in Tanzania incorporated an image of a Baobab tree and a widely known local idiom, ‘Together we can hug the Baobab tree’ on the calendar and the small plastic pillbox given to the patient receiving HIV care [143]. The Baobab tree, also known as the ‘tree of life’ locally, was a positive image known by residents where the trees are numerous in the community. Similarly, a social marketing campaign intervention in the USA involved utilised graphic novels using a group of inspirational superheroes called ‘The Undetectables’ as a priming stimulus to engage and motivate people living with HIV (PLWHIV) to manage their ART adherence [144]. Other examples included personalised reminder messages, cues, or feedback to inform the individual of their adherence or remind them of the medication times via text messages or phone calls (Table 2).

### Affect

Seven HIV prevention studies used the affect nudge. For example, individuals were invited to celebrate testing negative HIV/STI with their favourite or an indulgent activity [139], or individuals were provided with positive automated feedback through an app and messages when set goals were achieved and with messages from peer mentors to motivate accessing testing services [149]. Some studies used negative emotions associated with increased risk perception of getting HIV to encourage healthy behaviours [99, 151].

Ten HIV management studies used the affect nudge. In one study, patients would receive congratulations for attending the clinic three consecutive on-time visits for HIV care, and they were given a sticker to proudly place on an interactive poster publicly displayed in the clinic to celebrate and acknowledge their achievement [143]. Another study involved a multi-component positive affect intervention for MSM who used drugs, eight core skills, and meditation exercises were delivered to them to increase positive affect among them and to improve their psychological adjustment to cope effectively with methamphetamine withdrawal, to reduce their HIV viral load ultimately through this mediation [152].

### Commitment

Twenty HIV prevention studies used commitment. Binding commitment contracts, which involved financial deposits made by participants, were used in some studies to encourage participants to attend clinics regularly for HIV testing services [139, 156, 157]. When participants underwent HIV testing regularly for the agreed commitment period, their deposits were returned with interest paid. Otherwise, if they failed to regularly attend the HIV prevention services, they forfeited their deposits and incurred financial loss. Alternatively, some studies targeted cognitive dissonance by implementing soft or non-binding commitment on the individuals, such as having participants express their intention to get HIV tested on a sheet of paper or selected a date and time on a calendar to attend the community health campaign for HIV prevention services [141, 160]. Other examples included commitments that provide participants with financial rewards or incentives, lottery tickets, or prize draws for meeting a certain specified goal or health behaviour in the future [161–163] (Table 2).

Thirty-two HIV management studies used commitment. Provided participants met a specified health outcome in the future, they were promised financial incentives such as vouchers [169], prize draws [170], and lottery prizes [171], or non-financial incentives such as mobile airtime [155, 184], and food [117, 185]. These incentives were provided to participants for health outcomes such as suppressing the viral load to a specified level (i.e. HIV RNA < 200 copies/mL) for a period of time, for example for 6 or 12 months [169–171], maintaining a high level of ART adherence [155], attending HIV management services regularly [117, 172, 185], and having linkage to HIV care within 1 month of positive HIV testing and visiting treatment services regularly [184, 186].

### Ego

Thirteen HIV prevention studies used ego. HIV prevention education programmes were used to increase self-efficacy, negotiation, and communication skills related to HIV risk behaviours and sexual relationships [31, 187, 188]. These programmes were usually paired with other training and programmes which taught financial knowledge and skills, such as tailored microenterprise training [187], financial education programmes [31, 158], and micro-business start-up grants [18, 188] (Table 2).

Twenty-three HIV management studies used ego. This included participants attending motivational interviewing sessions with a psychologist or a specialist nurse [169, 182, 194], and participants regularly receiving carefully crafted motivational messages to target their ego to influence their health behaviours, for example messages such as ‘Stay strong!’, ‘Have courage!’, or ‘Don’t give up!’ [147, 155]. A study aimed to evoke positive emotions in PLWHIV for maintaining their HIV care and management, for example by providing them with opportunities to participate in non-medical-related leisure activities, such as massage therapy and beauty consultancy, alongside their HIV intervention sessions [146]. Other examples involved participants receiving HIV education and cognitive behavioural skills-building sessions to affect their ego through empowering them with HIV knowledge and management skills to look after their health and wellbeing (Table 2).

## Discussion

We contribute to the literature by synthesizing the range of HIV prevention and management interventions which can be described by behavioural economic principles. To the best of our knowledge, our study is the first of its kind to use the MINDSPACE framework for describing HIV-related interventions. A variety of study designs were used that took place in range of settings, e.g. from the controlled environment of a clinical trial to quasi-experimental designs to qualitative interviews. Most interventions relied heavily on financial incentives to encourage behaviour change and thus most of the MINDSPACE effects are potentially underutilised. Future HIV prevention and management interventions could consider incorporating more MINDSPACE components and evaluate their potential to optimise the interventions’ effectiveness.

Using behavioural economics to inform interventions can benefit the HIV sector by providing a framework to support micro- and macro-level decision-making and improve health outcomes. Based on our review, we observe that the different MINDSPACE effects are already in use in multiple HIV prevention and management interventions. This demonstrates the feasibility for implementing these nudge effects. However, most effects are potentially underutilised. There could be greater use of nudges, for example implementing a default opt-out approach for HIV testing [41, 42, 133] or utilizing social norms by sending text messages to inform participants of their medication adherence level and adherence level of their peers [196]. We recommend the use of the MINDSPACE framework to consider whether the range of effects could be used in the design of an intervention. It is also important to note more than one effect can be used in the same intervention, which may further optimise the intervention’s effectiveness [12].

There can be disadvantages to using nudges in the design of HIV interventions. First, the effectiveness of nudge interventions are often context-dependent [197]. Additionally, there is little information about long-term behavioural changes resulting from these nudge interventions [198]. Additionally, the effects of nudges may weaken when more widely used, as individuals could become more aware of the intended effect and of their own decision-making biases [199]. Third, marginalised populations with limited resources may be at higher risk of being negatively impacted by health policies and interventions using nudges, such as default, because they may not be able to opt out without significant burden or cost [200].

Our study should be read in light of some limitations. First, we limited our search to four databases and English publications and may have missed other relevant literature. However, we aimed to collate examples of how behavioural economic principles were applied in the HIV sector and not provide an exhaustive list of all relevant studies. Second, it was beyond the scope of this paper to explore the effectiveness of the MINDSPACE interventions, which we will explore in a future publication.

There are several lessons for the future application of behavioural economics in the design of HIV prevention and management interventions. First, we found interesting examples of how nudges are applied, but we must be wary of one-size-fits-all nudges. Each nudge must be evaluated and carefully adapted to the local cultural context. Second, using a framework, like MINDSPACE, helps consider how nudges could be applied to help people go with the flow of their automatic (system 1) decision-making patterns. Third, as nudge interventions move beyond academic interest to practical applications, they need to demonstrate and communicate its impact, particularly evaluating the value of multifaceted behavioural solutions. Finally, MINDSPACE centred interventions should be aligned with the individual’s and the community’s existing identity and goals to make the interventions acceptable and successful. Importantly, when designing MINDSPACE-centred interventions, its impact on the vulnerable subset of target populations should be considered foremostly to ensure fairness and equity, and that the interventions remain a nudge as opposed to being coercive and involuntary. The results of rigorous field evaluations of interventions should inform the design and implementation of future programmes to have optimal impact of the nudge interventions on target populations.

## Conclusion

The key influences described by the MINDSPACE framework can describe aspects of HIV prevention and management interventions. Our study indicates that Messenger, Incentive, and Commitment were the most frequently applied nudges. Therefore, there is an opportunity for future interventions to explore the use of other nudges (e.g. default and priming) to gather further evidence to understand the feasibility and value of applying nudges in HIV prevention and management strategies. Further research, specifically about long-term behavioural changes regarding HIV health outcomes due to nudge interventions, is also warranted.

## Supplementary Information

Below is the link to the electronic supplementary material.

Supplementary file1 (DOCX 138 KB)

## Data Availability

All relevant data are presented in the manuscript and online supplementary materials. Any further details can be obtained by contacting the corresponding author.
