# Supplementary material for: Applying Behavioural Insights to HIV Prevention and Management: a Scoping Review
Source: Curr HIV/AIDS Rep. 2022 Aug 5;19(5):358–74. doi: 10.1007/s11904-022-00615-z (PMC9508055; doi:10.1007/s11904-022-00615-z)
Supplement: Supplementary file 1 — Supplementary file1 (DOCX 138 KB) [file 11904_2022_615_MOESM1_ESM.docx]

**Appendix**

**Supplementary Table 1: search terms used in the search strategy in the scoping review**

| **HIV/AIDS related terms** | **HIV Prevention and Management related terms** | **Behavioral economics related terms** | **MINDSPACE related terms** |
| --- | --- | --- | --- |
| HIV/AIDS  HIV + positive  HIV + negative  Human immunodeficiency virus  HIV + virus  HIV + infect* (infection/infected)  HIV + risk  HIV 1  HIV 2  AIDS  Acquired immunodeficiency syndrome  People + living + with + HIV  PLWHIV  PWHIV | Antiviral+ therap*  ART  Antiretroviral  Antiretroviral + treatment*  Antiretroviral + HIV treatment*  Antiretroviral + medication*  Antiretroviral + drug*  Antiretroviral + adherence  Antiretroviral + compliance  Antiretroviral + agent*  Medication + adherence  Medication + compliance  PrEP  Pre-exposure prophylaxis  PEP  Post-exposure prophylaxis  HIV + medication  HIV + prevention  HIV + prevention + intervention*  HIV + infection + therapy  HAART  Highly + active + antiretroviral + therapy  TasP  Treatment + as + Prevention  U=U  Undetectable = untransmittable  Testing  HIV self-testing  Condom* | Contingency + management  Incentiv*  Cash + incentive* (incentive/s)  Cash + payment* (+ HIV prevention)  Financial + incentiv*  Economic + incentiv*  Monetary + incentiv*  Financial + reward*  Reinforcement*  Monetary + reinforcement*  Financial + reinforcement*  Cash + reinforcement*  Token + reinforcement*  Voucher + reinforcement*  Economic + reinforcement*  Payment*  Monetary  Behavioral + economics  Behavioural + economics  Psychology + economics  Behavioral + insight*  Behavioural + insight*  Behavioral + change*  Behavioural + change*  Nudg*  Conditional cash transfer*  Conditional + economic incentiv*  Loss + aversion  Voucher + incentiv*  Voucher + reinforcement*  Choice architecture  Choice + intervention*  Cognitive + bias  Confirmation + bias  Lottery based incentiv*  Framing  Intervention*  Motivat*  Entic*  Induc* | Messenger  Incentives  Norms  Defaults  Salience  Priming  Affect  Commitment  Ego |

- * denotes multiple variants of the term (For example: plural or adjective variant)
- + indicates the terms were used as a combination

**Supplementary Table 2: HIV prevention studies found in the scoping review**

| **Reference** | **Country** | **Study design** | **Population** |
| --- | --- | --- | --- |
| (1) | Australia | Mixed methods | Medical staff |
| (2) | Australia | Cross-sectional | Overseas-born MSM in Australia |
| (3) | Botswana | Cohort | Women who utilized antenatal clinic |
| (4) | Canada | Qualitative | Health care or service provider, worked with people at risk or living with HIV |
| (5) | China | RCT | Age 18+, MSM, biological male |
| (6) | China | Cross sectional | Age 18+, general population |
| (7) | China | Cross sectional | Age 16+ MSM in China |
| (8) | China | Cross sectional | Age 18+, MSM |
| (9) | Ecuador | RCT | Age 18+, general population |
| (10) | El Salvador | Time series | Age 17 +, peer crack users, in San Salvador |
| (11) | Guyana | Cross sectional | Age 18+, general population |
| (12) | India | Cross sectional | Age 18 +, MSM |
| (13) | India | RCT | Age 18+, street based FSW |
| (14) | India | Cohort study | Age 18+ Injecting drug users, residing in or around Delhi |
| (15) | Ireland | Cross sectional | Age 18+, emergency dept. patients |
| (16) | Kenya | Qualitative | Health care providers |
| (17) | Kenya | RCT | Age 15-24, female |
| (18) | Kenya | RCT | LWHIV, female primary caregivers and had child(ren) ≤12 years and of unknown HIV status |
| (19) | Kenya | RCT | Adults living with HIV, with children 0–12 years of unknown HIV status |
| (20) | Kenya | RCT | Age 25-49, uncircumcised men |
| (21) | Kenya | RCT | Caregivers living with HIV |
| (22) | Kenya | Qualitative | Age 18+, living with HIV, adult caregivers and had at least one child < 13 years with an unknown HIV status |
| (23) | Malawi | RCT | Age 18+, women attending antenatal clinic with male, not already taking ART (ANC cohort)  Age 18+, women with newly diagnosed HIV during routine clinic HIV testing (Index cohort) |
| (24) | Malawi | RCT | Age 18+, pregnant women with male partner not known to be on ART |
| (25) | Malawi | Qualitative | Age 18+, women attending antenatal clinic or having a pregnant partner attending the clinic (men) |
| (26) | Malawi | Qualitative | Ever-married women and their husbands and Adolescents (ages 15–24) |
| (27) | Malawi | RCT | Ever-married women and their husbands and Adolescents (ages 15–24) |
| (28) | Malawi | Qualitative | Ever-married women and their husbands and Adolescents (ages 15–24) |
| (29) | South Africa | RCT | Age 18-40, women presenting for post-abortion family planning services |
| (30) | South Africa | RCT | Age 15+, general population |
| (31) | South Africa | RCT | Age 15+, male |
| (32) | South Africa | Cross sectional | Employees at independent auto supplier companies |
| (33) | Tanzania | Mixed method, evaluation | 7 pairs of facilities were selected from 17 available facilities in three regions |
| (34) | Tanzania | RCT | Age 18-30, males and females and their spouses starting at age 16 years and potentially older than 30 years, residing in one of ten villages |
| (35) | Tanzania | Qualitative | Age 15-24, female, not currently enrolled and have attended less than 10 days of school in the last 3 months |
| (36) | Tanzania | RCT | Age 18+, male |
| (37) | Thailand | Cross sectional | Gay men and other MSM |
| (38) | Uganda | RCT | Age 18-59, HIV negative and engaged in at least one risk factor in the previous 12 months |
| (39) | Uganda | RCT | Age 18-59, HIV negative |
| (40) | Uganda | RCT | Age 18+, male |
| (41) | Uganda | Qualitative study | Age 18+, male |
| (42) | Uganda | Cross sectional study | Age 10-17, lost at least one biological parent to AIDS, were registered in the last two years of primary school. Primary guardians (age 18 +) were also recruited. |
| (43) | Uganda | RCT | AIDS orphaned youth, in primary school—just prior to the transition to secondary school |
| (44) | USA | Mixed method | Age 18+, female, reported STI risk in previous four months |
| (45) | USA | RCT | Age 18-49, HIV negative, justice involved MSM and transgender women, with substance use disorders |
| (46) | USA | RCT | Age 18-24, African American, living in Baltimore, having experienced at least one episode of homelessness in the last 12 months |
| (47) | USA | Multi-component, multiple methods | HIV negative and PLWHIV |
| (48) | USA | Time-samples design | Age 16+, HIV negative, emergency dept. patients |
| (49) | USA | Qualitative interviews | Age 18+, Engaged in frequent cocaine or heroin injection in the past 3 months, residing in Baltimore, USA |
| (50) | USA | RCT | Age 18+, Latinx MSM or TGW, fluent in English or Spanish |
| (51) | USA | Cohort | Age 18+, Latinx MSM or TGW, fluent in English or Spanish |
| (52) | USA | Cohort | Age 18+, HIV negative, Latinx sexual minority men and TGW, all staff enrolling participants into the study |
| (53) | USA | Randomized trial | Age 30-60, African American or black residents of Oakland, CA |
| (54) | USA | Clinical trial | Age 18-30, Male at birth, self-report having sex with men in the last 6 months, self-report being currently prescribed and taking PrEP for HIV prevention |
| (55) | USA | RCT | Age 13–64, HIV negative, emergency dept. patients |
| (56) | USA | RCT | Age 13–64, HIV negative, emergency dept. patients |
| (57) | USA | RCT | Age 13–64, HIV negative, emergency dept. patients, cost utility analysis |
| (58) | USA | Cohort | Age 15+, medically stable, emergency dept. patient |
| (59) | Zambia | Cohort | Age 18+, VMMC clients |
| (60) | Zimbabwe | RCT | Age 8-17, in Harare |
| (61) | Zimbabwe | Cohort | Age 15-24, adolescent girls and young women and age 15-29 VMMC in their male partners. |
| (62) | Zimbabwe | RCT | Population level (44 out of a possible 254 wards) |
| (63) | Zimbabwe | RCT | Population level (68 rural communities in four districts) |
| (64) | Zimbabwe | RCT | Age 15-29, HIV negative, male, not previously circumcised, residing in Manicaland province |

**Abbreviations:** VMMC- Voluntary medical male circumcision; MSM- Men who have sex with men; TGW- Transgender women; PLWHIV- People living with HIV; ART- Anti-retroviral therapy

**Supplementary Table 3: HIV Management studies found in the scoping review**

| **Reference** | **Country** | **Study Design** | **Population** |
| --- | --- | --- | --- |
| (65) | Canada | Qualitative | Health care providers, work experience with PLWHIV |
| (66) | Congo | RCT | PLWHIV, women, pregnant |
| (67) | Congo | RCT | PLWHIV, women, pregnant, recent HIV diagnosis |
| (68) | England | Cohort study | Age 16-25, PLWHIV, youth, HIV perinatally acquired |
| (69) | Ghana | Mixed method | Age 13-18, PLWHIV, adolescents, on ART |
| (70) | Kenya | RCT | Age 15-24, female, children and young adults |
| (71) | Mozambique | Mixed method | Age 18+, PLWHIV |
| (72) | Mozambique | Process evaluation | Age 18+, PLWHIV |
| (73) | Nigeria | Mixed method | Age 10-19, PLWHIV, children and young adults |
| (74) | South Africa | RCT | Age 18+, PLWHIV, men |
| (75) | South Africa | Cohort study | Age 18+, PLWHIV, women, pregnant |
| (30) | South Africa | RCT | Age 15+, households within AHRI HIV Surveillance |
| (76) | South Africa | RCT | Age 18+, PLWHIV |
| (77) | South Africa | Qualitative | Age 18+, PLWHIV, ART-naïve |
| (78) | Swaziland | RCT | Age 18+, PLWHIV, recent HIV diagnosis |
| (79) | Tanzania | Mixed method | Age 18+, PLWHIV, on ART, food insecure |
| (80) | Tanzania | RCT | Age 18+, PLWHIV, on ART, food insecure |
| (81) | Tanzania | Mixed method | Age 18+, PLWHIV, on ART |
| (82) | Tanzania | RCT | Age 18+, PLWHIV, on ART, food insecure |
| (83) | Tanzania | Cohort study | Age 18+, PLWHIV, receiving HIV care |
| (84) | Tanzania | RCT; Impact Evaluation | PLWHIV, on ART |
| (85) | Thailand | Cross sectional | Age 14+, MSM |
| (86) | Uganda | RCT | Age 18+, PLWHIV, on ART |
| (87) | Uganda | RCT | Age 18+, PLWHIV, on ARVs |
| (88) | Uganda | Mixed methods | Age15-24, PLWHIV, children and young adults, on ART |
| (89) | Uganda | RCT | Age 15-24, children and young adults |
| (90) | Uganda | Mixed methods | Age 17-24, PLWHIV, children and young adults |
| (91) | Uganda | Cross-sectional | Age 10-17, adolescents;  Age 18+, primary caregiver |
| (92) | Uganda | Cohort study | PLWHIV, general population, on ART or ART-naïve |
| (93) | Uganda | Cohort study | Age 10-16, PLWHIV, adolescents |
| (94) | Uganda | RCT | Age 18+, PLWHIV |
| (95) | USA | Cohort study? | PLWHIV, homeless |
| (96) | USA | Qualitative | Age 34-50, PLWHIV, receive HIV care |
| (97) | USA | RCT | Age 18+, PLWHIV, on ART |
| (98) | USA | Cohort study? | Age 13+, PLWHIV, general population |
| (99) | USA | RCT | Age 18+, PLWHIV, receive HIV care, substance use |
| (100) | USA | Time-series | PLWHIV, receive HIV care |
| (101) | USA | RCT | PLWHIV, receive HIV care |
| (102) | USA | Cohort study | PLWHIV, on ART |
| (103) | USA | Program impact evaluation | PLWHIV |
| (104) | USA | Cohort study | Emergency dept. patients, high risk of HIV infection |
| (105) | USA | RCT | Age 18+, PLWHIV, MSM, on ART, substance use |
| (106) | USA | RCT | PLWHIV, substance use |
| (107) | USA | Qualitative | PLWHIV |
| (108) | USA | Economic evaluation | PLWHIV, general population, receive HIV care |
| (109) | USA | RCT | PLWHIV, general population |
| (110) | USA | Qualitative | Clinic staff, work experience with PLWHIV |
| (111) | USA | RCT | Age 18+, PLWHIV, MSM |
| (112) | USA | Cohort study | PLWHIV, MSM |
| (113) | USA | Mixed method | PLWHIV, socially vulnerable (homelessness, substance use, mental illness) |
| (114) | USA | Qualitative | PLWHIV, socially vulnerable (homelessness, substance use, mental illness) |
| (115) | USA | Qualitative | PLWHIV, on ART |
| (116) | USA | RCT? | PLWHIV, women |
| (117) | USA | RCT | Age 18+, PLWHIV, general population |
| (118) | USA | RCT | Age 18+, PLWHIV, women (cisgender or transgender) |
| (119) | USA | Qualitative | PLWHIV, general population |
| (120) | USA | RCT | PLWHIV, on ART, substance use |
| (121) | USA | Qualitative | PLWHIV, recent HIV diagnosis or currently out of HIV care for 1 year |
| (122) | USA | RCT | Age 18+, PLWHIV, recent HIV diagnosis |
| (123) | USA | RCT | Age 12-20, PLWHIV, children and young adults, on ART, non-adherent to medication |
| (124) | USA | Qualitative | Age 14+, PLWHIV |
| (125) | n/a | Review | PLWHIV, mothers |

**Abbreviations:** MSM- Men who have sex with men; PLWHIV- People living with HIV; ART- Anti-retroviral therapy; ARV- Antiretrovirals; AHRI- Africa Health Research Institute

**REFERENCES**

1. Leidel S, Leslie G, Boldy D, Davies A, Girdler S. 'We didn't have to dance around it': opt-out HIV testing among homeless and marginalised patients. Aust J Prim Health. 2017;23(3):278-83.

2. Ong JJ, Chow EPF, Read D, Taj U, Lee D, Vlaev I. Nudgeathons to control HIV: designing strategies using behavioural economics. AIDS. 2020;34(15):2337-40.

3. Creek TL, Ntumy R, Seipone K, Smith M, Mogodi M, Smit M, et al. Successful introduction of routine opt-out HIV testing in antenatal care in Botswana. J Acquir Immune Defic Syndr. 2007;45(1):102-7.

4. Gagnon M, Guta A, Upshur R, Murray SJ, Bungay V. "It gets people through the door": a qualitative case study of the use of incentives in the care of people at risk or living with HIV in British Columbia, Canada. BMC Med Ethics. 2020;21(1):105.

5. Lu Y, Ni Y, Li X, He X, Huang S, Zhou Y, et al. Monetary incentives and peer referral in promoting digital network-based secondary distribution of HIV self-testing among men who have sex with men in China: study protocol for a three-arm randomized controlled trial. BMC Public Health. 2020;20(1):911.

6. Tam G, Wong SYS. A Cross-Sectional Study Comparing Two Opt-Out HIV Testing Strategies in the Out-Patient Setting. Front Public Health. 2021;9(664494):664494.

7. Wang C, Tucker JD, Liu C, Zheng H, Tang W, Ling L. Condom use social norms and self-efficacy with different kinds of male partners among Chinese men who have sex with men: results from an online survey. BMC Public Health. 2018;18(1):1175.

8. Zou H, Wu Z, Yu J, Li M, Ablimit M, Li F, et al. Internet-facilitated, voluntary counseling and testing (VCT) clinic-based HIV testing among men who have sex with men in China. PLoS One. 2013;8(2):e51919.

9. Macis M, Grunauer M, Gutierrez E, Izurieta R, Phan P, Reina Ortiz M, et al. Using Incentives and Nudging to Improve Non-Targeted HIV Testing in Ecuador: A Randomized Trial. AIDS Behav. 2021;25(8):2542-50.

10. Glasman LR, Dickson-Gomez J, Lechuga J, Tarima S, Bodnar G, de Mendoza LR. Using Peer-Referral Chains with Incentives to Promote HIV Testing and Identify Undiagnosed HIV Infections Among Crack Users in San Salvador. AIDS Behav. 2016;20(6):1236-43.

11. Christensen A, Russ S, Rambaran N, Wright SW. Patient perspectives on opt-out HIV screening in a Guyanese emergency department. Int Health. 2012;4(3):185-91.

12. Das A, George B, Ranebennur V, Parthasarathy MR, Shreenivas GS, Todankar P, et al. Getting to the First 90: Incentivized Peer Mobilizers Promote HIV Testing Services to Men Who Have Sex With Men Using Social Media in Mumbai, India. Glob Health Sci Pract. 2019;7(3):469-77.

13. Sherman SG, Srikrishnan AK, Rivett KA, Liu SH, Solomon S, Celentano DD. Acceptability of a microenterprise intervention among female sex workers in Chennai, India. AIDS Behav. 2010;14(3):649-57.

14. Tun W, Sebastian MP, Sharma V, Madan I, Souidi S, Lewis D, et al. Strategies for recruiting injection drug users for HIV prevention services in Delhi, India. Harm Reduct J. 2013;10:16.

15. O'Connell S, Lillis D, Cotter A, O'Dea S, Tuite H, Fleming C, et al. Opt-Out Panel Testing for HIV, Hepatitis B and Hepatitis C in an Urban Emergency Department: A Pilot Study. PLoS One. 2016;11(3):e0150546.

16. Atkins DL, Wagner AD, Zhang J, Njuguna IN, Neary J, Omondi VO, et al. Brief Report: Use of the Consolidated Framework for Implementation Research (CFIR) to Characterize Health Care Workers' Perspectives on Financial Incentives to Increase Pediatric HIV Testing. J Acquir Immune Defic Syndr. 2020;84(1):e1-e6.

17. Inwani I, Chhun N, Agot K, Cleland CM, Buttolph J, Thirumurthy H, et al. High-Yield HIV Testing, Facilitated Linkage to Care, and Prevention for Female Youth in Kenya (GIRLS Study): Implementation Science Protocol for a Priority Population. JMIR Res Protoc. 2017;6(12):e179.

18. Njuguna IN, Wagner AD, Omondi VO, Otieno VA, Neary J, Bosire R, et al. Financial Incentives for Pediatric HIV Testing in Kenya. Pediatr Infect Dis J. 2018;37(11):1142-4.

19. Njuguna IN, Wagner AD, Neary J, Omondi VO, Otieno VA, Orimba A, et al. Financial incentives to increase pediatric HIV testing: a randomized trial. AIDS. 2021;35(1):125-30.

20. Thirumurthy H, Masters SH, Rao S, Bronson MA, Lanham M, Omanga E, et al. Effect of providing conditional economic compensation on uptake of voluntary medical male circumcision in Kenya: a randomized clinical trial. JAMA. 2014;312(7):703-11.

21. Wagner AD, Shah SK, Njuguna IN, Porter KM, Neary J, Maleche-Obimbo E, et al. Financial Incentives to Motivate Pediatric HIV Testing-Assessing the Potential for Coercion, Inducement, and Voluntariness. J Acquir Immune Defic Syndr. 2018;78(3):e15-e8.

22. Zhang J, Atkins DL, Wagner AD, Njuguna IN, Neary J, Omondi VO, et al. Financial Incentives for Pediatric HIV Testing (FIT): Caregiver Insights on Incentive Mechanisms, Focus Populations, and Acceptability for Programmatic Scale Up. AIDS Behav. 2021;25(9):2661-8.

23. Choko AT, Fielding K, Johnson CC, Kumwenda MK, Chilongosi R, Baggaley RC, et al. Partner-delivered HIV self-test kits with and without financial incentives in antenatal care and index patients with HIV in Malawi: a three-arm, cluster-randomised controlled trial. Lancet Glob Health. 2021;9(7):e977-e88.

24. Choko AT, Corbett EL, Stallard N, Maheswaran H, Lepine A, Johnson CC, et al. HIV self-testing alone or with additional interventions, including financial incentives, and linkage to care or prevention among male partners of antenatal care clinic attendees in Malawi: An adaptive multi-arm, multi-stage cluster randomised trial. PLoS Med. 2019;16(1):e1002719.

25. Choko AT, Kumwenda MK, Johnson CC, Sakala DW, Chikalipo MC, Fielding K, et al. Acceptability of woman-delivered HIV self-testing to the male partner, and additional interventions: a qualitative study of antenatal care participants in Malawi. J Int AIDS Soc. 2017;20(1):21610.

26. Godlonton S, Thornton R. Peer effects in learning HIV results. J Dev Econ. 2012;97(1):118-29.

27. Kohler HP, Thornton R. Conditional Cash Transfers and HIV/AIDS Prevention: Unconditionally Promising? World Bank Econ Rev. 2012;26(2):165-90.

28. Thornton RL. The Demand for, and Impact of, Learning HIV Status. Am Econ Rev. 2008;98(5):1829-63.

29. Galarraga O, Harries J, Maughan-Brown B, Cooper D, Short SE, Lurie MN, et al. The Empower Nudge lottery to increase dual protection use: a proof-of-concept randomised pilot trial in South Africa. Reprod Health Matters. 2018;26(52):1510701.

30. Mathenjwa T, Kim HY, Zuma T, Shahmanesh M, Seeley J, Matthews P, et al. Home-based intervention to test and start (HITS) protocol: a cluster-randomized controlled trial to reduce HIV-related mortality in men and HIV incidence in women through increased coverage of HIV treatment. BMC Public Health. 2019;19(1):969.

31. Tanser FC, Kim HY, Mathenjwa T, Shahmanesh M, Seeley J, Matthews P, et al. Home-Based Intervention to Test and Start (HITS): a community-randomized controlled trial to increase HIV testing uptake among men in rural South Africa. J Int AIDS Soc. 2021;24(2):e25665.

32. Weihs M, Meyer-Weitz A, Baasner-Weihs F. The influence of lotteries on employees' workplace HIV testing behaviour. Afr J AIDS Res. 2018;17(1):9-21.

33. Bazant E, Mahler H, Machaku M, Lemwayi R, Kulindwa Y, Gisenge Lija J, et al. A Randomized Evaluation of a Demand Creation Lottery for Voluntary Medical Male Circumcision Among Adults in Tanzania. J Acquir Immune Defic Syndr. 2016;72 Suppl 4:S280-7.

34. de Walque D, Dow WH, Nathan R, Abdul R, Abilahi F, Gong E, et al. Incentivising safe sex: a randomised trial of conditional cash transfers for HIV and sexually transmitted infection prevention in rural Tanzania. BMJ Open. 2012;2:e000747.

35. Gichane MW, Wamoyi J, Atkins K, Balvanz P, Maman S, Majani E, et al. The influence of cash transfers on engagement in transactional sex and partner choice among adolescent girls and young women in Northwest Tanzania. Cult Health Sex. 2020:1-15.

36. Ong JJ, Neke N, Wambura M, Kuringe E, Grund JM, Plotkin M, et al. Use of Lotteries for the Promotion of Voluntary Medical Male Circumcision Service: A Discrete-Choice Experiment among Adult Men in Tanzania. Med Decis Making. 2019;39(4):474-85.

37. Anand T, Nitpolprasert C, Ananworanich J, Pakam C, Nonenoy S, Jantarapakde J, et al. Innovative strategies using communications technologies to engage gay men and other men who have sex with men into early HIV testing and treatment in Thailand. J Virus Erad. 2015;1(2):111-5.

38. Chamie G, Kwarisiima D, Ndyabakira A, Marson K, Camlin CS, Havlir DV, et al. Financial incentives and deposit contracts to promote HIV retesting in Uganda: A randomized trial. PLoS Med. 2021;18(5):e1003630.

39. Chamie G, Ndyabakira A, Marson KG, Emperador DM, Kamya MR, Havlir DV, et al. A pilot randomized trial of incentive strategies to promote HIV retesting in rural Uganda. PLoS One. 2020;15(5):e0233600.

40. Kavanagh NM, Schaffer EM, Ndyabakira A, Marson K, Havlir DV, Kamya MR, et al. Planning prompts to promote uptake of HIV services among men: a randomised trial in rural Uganda. BMJ Glob Health. 2020;5(11):11.

41. Ndyabakira A, Getahun M, Byamukama A, Emperador D, Kabageni S, Marson K, et al. Leveraging incentives to increase HIV testing uptake among men: qualitative insights from rural Uganda. BMC Public Health. 2019;19(1):1763.

42. Mayo-Wilson LJ, Ssewamala FM. Financial and Behavioral Economic Factors Associated With HIV Testing In AIDS-Affected Adolescents in Uganda: A Cross-Sectional Analysis. J Health Care Poor Underserved. 2019;30(1):339-57.

43. Ssewamala FM, Ismayilova L, McKay M, Sperber E, Bannon W, Jr., Alicea S. Gender and the effects of an economic empowerment program on attitudes toward sexual risk-taking among AIDS-orphaned adolescent youth in Uganda. J Adolesc Health. 2010;46(4):372-8.

44. Beadnell B, Baker S, Knox K, Stielstra S, Morrison DM, Degooyer E, et al. The influence of psychosocial difficulties on women's attrition in an HIV/STD prevention program. AIDS Care. 2003;15(6):807-20.

45. Edwards GG, Reback CJ, Cunningham WE, Hilliard CL, McWells C, Mukherjee S, et al. Mobile-Enhanced Prevention Support Study for Men Who Have Sex With Men and Transgender Women Leaving Jail: Protocol for a Randomized Controlled Trial. JMIR Res Protoc. 2020;9(9):e18106.

46. Mayo-Wilson LJ, Glass NE, Ssewamala FM, Linnemayr S, Coleman J, Timbo F, et al. Microenterprise intervention to reduce sexual risk behaviors and increase employment and HIV preventive practices in economically-vulnerable African-American young adults (EMERGE): protocol for a feasibility randomized clinical trial. Trials. 2019;20(1):439.

47. Gamble T, Branson B, Donnell D, Hall HI, King G, Cutler B, et al. Design of the HPTN 065 (TLC-Plus) study: A study to evaluate the feasibility of an enhanced test, link-to-care, plus treat approach for HIV prevention in the United States. Clin Trials. 2017;14(4):322-32.

48. Haukoos JS, Hopkins E, Conroy AA, Silverman M, Byyny RL, Eisert S, et al. Routine opt-out rapid HIV screening and detection of HIV infection in emergency department patients. JAMA. 2010;304(3):284-92.

49. Latkin CA, Forman V, Knowlton A, Sherman S. Norms, social networks, and HIV-related risk behaviors among urban disadvantaged drug users. Social Science & Medicine. 2003;56(3):465-76.

50. Linnemayr S, MacCarthy S, Kim A, Giguere R, Carballo-Dieguez A, Barreras JL. Behavioral economics-based incentives supported by mobile technology on HIV knowledge and testing frequency among Latino/a men who have sex with men and transgender women: Protocol for a randomized pilot study to test intervention feasibility and acceptability. Trials. 2018;19(1):540.

51. MacCarthy S, Wagner Z, Barreras JL, Kim A, Menodza-Graf AC, Giguere R, et al. Brief Report: Using Behavioral Economics to Increase HIV Knowledge and Testing Among Latinx Sexual Minority Men and Transgender Women: A Quasi-Experimental Pilot Study. J Acquir Immune Defic Syndr. 2020;85(2):189-94.

52. MacCarthy S, Mendoza-Graf A, Wagner Z, J LB, Kim A, Giguere R, et al. The acceptability and feasibility of a pilot study examining the impact of a mobile technology-based intervention informed by behavioral economics to improve HIV knowledge and testing frequency among Latinx sexual minority men and transgender women. BMC Public Health. 2021;21(1):341.

53. McCoy SI, Shiu K, Martz TE, Smith CD, Mattox L, Gluth DR, et al. Improving the efficiency of HIV testing with peer recruitment, financial incentives, and the involvement of persons living with HIV infection. J Acquir Immune Defic Syndr. 2013;63(2):e56-63.

54. Mitchell JT, LeGrand S, Hightow-Weidman LB, McKellar MS, Kashuba AD, Cottrell M, et al. Smartphone-Based Contingency Management Intervention to Improve Pre-Exposure Prophylaxis Adherence: Pilot Trial. JMIR Mhealth Uhealth. 2018;6(9):e10456.

55. Montoy JCC, Dow WH, Kaplan BC. Cash incentives versus defaults for HIV testing: A randomized clinical trial. PLoS One. 2018;13(7):e0199833.

56. Montoy JC, Dow, W. H., & Kaplan, B. C. Patient choice in opt-in, active choice, and opt-out HIV screening: randomized clinical trial. BMJ. 2016;532(4):h6895.

57. Wagner Z, Montoy JCC, Drabo EF, Dow WH. Incentives Versus Defaults: Cost-Effectiveness of Behavioral Approaches for HIV Screening. AIDS Behav. 2020;24(2):379-86.

58. White DA, Scribner AN, Vahidnia F, Dideum PJ, Gordon DM, Frazee BW, et al. HIV screening in an urban emergency department: comparison of screening using an opt-in versus an opt-out approach. Ann Emerg Med. 2011;58(1 Suppl 1):S89-95.

59. Zanolini A, Bolton C, Lyabola LL, Phiri G, Samona A, Kaonga A, et al. Feasibility and Effectiveness of a Peer Referral Incentive Intervention to Promote Male Circumcision Uptake in Zambia. J Acquir Immune Defic Syndr. 2016;72 Suppl 4:S257-63.

60. Kranzer K, Simms V, Bandason T, Dauya E, McHugh G, Munyati S, et al. Economic incentives for HIV testing by adolescents in Zimbabwe: a randomised controlled trial. Lancet HIV. 2018;5(2):e79-e86.

61. Moorhouse L, Schaefer R, Thomas R, Nyamukapa C, Skovdal M, Hallett TB, et al. Application of the HIV prevention cascade to identify, develop and evaluate interventions to improve use of prevention methods: examples from a study in east Zimbabwe. J Int AIDS Soc. 2019;22 Suppl 4:e25309.

62. Sibanda EL, Neuman M, Tumushime M, Mangenah C, Hatzold K, Watadzaushe C, et al. Community-based HIV self-testing: a cluster-randomised trial of supply-side financial incentives and time-trend analysis of linkage to antiretroviral therapy in Zimbabwe. BMJ Glob Health. 2021;6(Suppl 4):07.

63. Sibanda EL, Tumushime M, Mufuka J, Mavedzenge SN, Gudukeya S, Bautista-Arredondo S, et al. Effect of non-monetary incentives on uptake of couples' counselling and testing among clients attending mobile HIV services in rural Zimbabwe: a cluster-randomised trial. Lancet Glob Health. 2017;5(9):e907-e15.

64. Thomas R, Skovdal M, Galizzi MM, Schaefer R, Moorhouse L, Nyamukapa C, et al. Improving risk perception and uptake of voluntary medical male circumcision with peer-education sessions and incentives, in Manicaland, East Zimbabwe: study protocol for a pilot randomised trial. Trials. 2020;21(1):108.

65. Gagnon M, Guta A, Upshur R, Murray SJ, Bungay V. "It gets people through the door": a qualitative case study of the use of incentives in the care of people at risk or living with HIV in British Columbia, Canada. BMC Medical Ethics. 2020;21(1):105.

66. Saleska JL, Turner AN, Gallo MF, Shoben A, Kawende B, Ravelomanana NLR, et al. Role of temporal discounting in a conditional cash transfer (CCT) intervention to improve engagement in the prevention of mother-to-child transmission (PMTCT) cascade. BMC Public Health. 2021;21(1):477.

67. Yotebieng M, Moracco KE, Thirumurthy H, Edmonds A, Tabala M, Kawende B, et al. Conditional Cash Transfers Improve Retention in PMTCT Services by Mitigating the Negative Effect of Not Having Money to Come to the Clinic. Journal of Acquired Immune Deficiency Syndromes: JAIDS. 2017;74(2):150-7.

68. Foster C, McDonald S, Frize G, Ayers S, Fidler S. "Payment by Results"--financial incentives and motivational interviewing, adherence interventions in young adults with perinatally acquired HIV-1 infection: a pilot program. AIDS Patient Care & Stds. 2014;28(1):28-32.

69. Galarraga O, Enimil A, Bosomtwe D, Cao W, Barker DH. Group-based economic incentives to improve adherence to antiretroviral therapy among youth living with HIV: safety and preliminary efficacy from a pilot trial. Vulnerable Children & Youth Studies. 2020;15(3):257-68.

70. Inwani I, Chhun N, Agot K, Cleland CM, Buttolph J, Thirumurthy H, et al. High-Yield HIV Testing, Facilitated Linkage to Care, and Prevention for Female Youth in Kenya (GIRLS Study): Implementation Science Protocol for a Priority Population. JMIR Research Protocols. 2017;6(12):e179.

71. Elul B, Lahuerta M, Abacassamo F, Lamb MR, Ahoua L, McNairy ML, et al. A combination strategy for enhancing linkage to and retention in HIV care among adults newly diagnosed with HIV in Mozambique: study protocol for a site-randomized implementation science study. BMC Infectious Diseases. 2014;14:549.

72. Sutton R, Lahuerta M, Abacassamo F, Ahoua L, Tomo M, Lamb MR, et al. Feasibility and Acceptability of Health Communication Interventions Within a Combination Intervention Strategy for Improving Linkage and Retention in HIV Care in Mozambique. Journal of Acquired Immune Deficiency Syndromes: JAIDS. 2017;74 Suppl 1:S29-S36.

73. Ekwunife OI, Ofomata CJ, Okafor CE, Anetoh MU, Kalu SO, Ele PU, et al. Cost-effectiveness and feasibility of conditional economic incentives and motivational interviewing to improve HIV health outcomes of adolescents living with HIV in Anambra State, Nigeria. BMC Health Services Research. 2021;21(1):685.

74. Barnabas RV, van Heerden A, McConnell M, Szpiro AA, Krows ML, Schaafsma TT, et al. Lottery incentives have short-term impact on ART initiation among men: results from a randomized pilot study. Journal of the International AIDS Society. 2020;23 Suppl 2:e25519.

75. Clouse K, Mongwenyana C, Musina M, Bokaba D, Long L, Maskew M, et al. Acceptability and feasibility of a financial incentive intervention to improve retention in HIV care among pregnant women in Johannesburg, South Africa. AIDS Care. 2018;30(4):453-60.

76. Maughan-Brown B, Smith P, Kuo C, Harrison A, Lurie MN, Bekker L-G, et al. A conditional economic incentive fails to improve linkage to care and antiretroviral therapy initiation among HIV-positive adults in Cape Town, South Africa. AIDS Patient Care and STDs. 2018;32(2):70-8.

77. Swartz A, Maughan-Brown B, Perera S, Harrison A, Kuo C, Lurie MN, et al. "The Money, It's OK but It's not OK": Patients' and Providers' Perceptions of the Acceptability of Cash Incentives for HIV Treatment Initiation in Cape Town, South Africa. AIDS & Behavior. 2021;20:20.

78. McNairy ML, Lamb MR, Gachuhi AB, Nuwagaba-Biribonwoha H, Burke S, Mazibuko S, et al. Effectiveness of a combination strategy for linkage and retention in adult HIV care in Swaziland: The Link4Health cluster randomized trial. PLoS Medicine / Public Library of Science. 2017;14(11):e1002420.

79. Czaicki NL, Mnyippembe A, Blodgett M, Njau P, McCoy SI. It helps me live, sends my children to school, and feeds me: a qualitative study of how food and cash incentives may improve adherence to treatment and care among adults living with HIV in Tanzania. AIDS Care. 2017;29(7):876-84.

80. Czaicki NL, Dow WH, Njau PF, McCoy SI. Do incentives undermine intrinsic motivation? Increases in intrinsic motivation within an incentive-based intervention for people living with HIV in Tanzania. PLoS ONE [Electronic Resource]. 2018;13(6):e0196616.

81. Fahey CA, Njau PF, Katabaro E, Mfaume RS, Ulenga N, Mwenda N, et al. Financial incentives to promote retention in care and viral suppression in adults with HIV initiating antiretroviral therapy in Tanzania: a three-arm randomised controlled trial. The Lancet HIV. 2020;7(11):e762-e71.

82. Fahey CA, Njau PF, Dow WH, Kapologwe NA, McCoy SI. Effects of short-term cash and food incentives on food insecurity and nutrition among HIV-infected adults in Tanzania. AIDS. 2019;33(3):515-24.

83. McCoy SI, Fahey C, Rao A, Kapologwe N, Njau PF, Bautista-Arredondo S. Pilot study of a multi-pronged intervention using social norms and priming to improve adherence to antiretroviral therapy and retention in care among adults living with HIV in Tanzania. PLoS ONE [Electronic Resource]. 2017;12(5):e0177394.

84. Packel L, Fahey C, Njau P, McCoy SI. Implementation Science Using Proctor's Framework and an Adaptation of the Multiphase Optimization Strategy: Optimizing a Financial Incentive Intervention for HIV Treatment Adherence in Tanzania. Journal of Acquired Immune Deficiency Syndromes: JAIDS. 2019;82 Suppl 3:S332-S8.

85. Anand T, Nitpolprasert C, Ananworanich J, Pakam C, Nonenoy S, Jantarapakde J, et al. Innovative strategies using communications technologies to engage gay men and other men who have sex with men into early HIV testing and treatment in Thailand. Journal of Virus Eradication. 2015;1(2):111-5.

86. Linnemayr S, Stecher C, Saya U, MacCarthy S, Wagner Z, Jennings L, et al. Behavioral Economics Incentives to Support HIV Treatment Adherence (BEST): Protocol for a randomized controlled trial in Uganda. Trials [Electronic Resource]. 2020;21(1):9.

87. Linnemayr S, Stecher C. Behavioral Economics Matters for HIV Research: The Impact of Behavioral Biases on Adherence to Antiretrovirals (ARVs). AIDS & Behavior. 2015;19(11):2069-75.

88. MacCarthy S, Wagner Z, Mendoza-Graf A, Gutierrez CI, Samba C, Birungi J, et al. A randomized controlled trial study of the acceptability, feasibility, and preliminary impact of SITA (SMS as an Incentive To Adhere): a mobile technology-based intervention informed by behavioral economics to improve ART adherence among youth in Uganda. BMC Infectious Diseases. 2020;20(1):173.

89. MacCarthy S, Mendoza-Graf A, Saya U, Samba C, Birungi J, Okoboi S, et al. Lessons learned from a mobile technology-based intervention informed by behavioral economics to improve ART adherence among youth in Uganda. AIDS Care. 2020;32(5):616-22.

90. MacCarthy S, Mendoza-Graf A, Huang H, Mukasa B, Linnemayr S. Supporting Adolescents to Adhere (SATA): Lessons learned from an intervention to achieve medication adherence targets among youth living with HIV in Uganda. Children & Youth Services Review. 2019;102:56-62.

91. Mayo-Wilson LJ, Ssewamala FM. Financial and Behavioral Economic Factors Associated With HIV Testing In AIDS-Affected Adolescents in Uganda: A Cross-Sectional Analysis. Journal of Health Care for the Poor & Underserved. 2019;30(1):339-57.

92. Siedner MJ, Santorino D, Lankowski AJ, Kanyesigye M, Bwana MB, Haberer JE, et al. A combination SMS and transportation reimbursement intervention to improve HIV care following abnormal CD4 test results in rural Uganda: A prospective observational cohort study. BMC Medicine. 2015;13(1).

93. Ssewamala FM, Sensoy Bahar O, Nabunya P, Thames AD, Neilands TB, Damulira C, et al. Suubi+Adherence-Round 2: A study protocol to examine the longitudinal HIV treatment adherence among youth living with HIV transitioning into young adulthood in Southern Uganda. BMC Public Health. 2021;21(1):179.

94. Thirumurthy H, Ndyabakira A, Marson K, Emperador D, Kamya M, Havlir D, et al. Financial incentives for achieving and maintaining viral suppression among HIV-positive adults in Uganda: a randomised controlled trial. The Lancet HIV. 2019;6(3):e155-e63.

95. Bamberger JD, Unick J, Klein P, Fraser M, Chesney M, Katz MH. Helping the urban poor stay with antiretroviral HIV drug therapy: Action Point services include counseling, medication, cash incentives, and even e-mail reminders. American Journal of Public Health. 2000;90(5):699-701.

96. Beima-Sofie K, Begnel ER, Golden MR, Moore A, Ramchandani M, Dombrowski JC. "It's Me as a Person, Not Me the Disease": Patient Perceptions of an HIV Care Model Designed to Engage Persons with Complex Needs. AIDS Patient Care & Stds. 2020;34(6):267-74.

97. Bien-Gund CH, Ho JI, Bair EF, Marcus N, Choi RJ, Szep Z, et al. Brief Report: Financial Incentives and Real-Time Adherence Monitoring to Promote Daily Adherence to HIV Treatment and Viral Suppression Among People Living With HIV: A Pilot Study. Journal of Acquired Immune Deficiency Syndromes: JAIDS. 2021;87(1):688-92.

98. Brantley AD, Burgess S, Bickham J, Wendell D, Gruber D. Using Financial Incentives to Improve Rates of Viral Suppression and Engagement in Care of Patients Receiving HIV Care at 3 Health Clinics in Louisiana: The Health Models Program, 2013-2016. Public Health Reports. 2018;133(2_suppl):75S-86S.

99. DeFulio A, Devoto A, Traxler H, Cosottile D, Fingerhood M, Nuzzo P, et al. Smartphone-based incentives for promoting adherence to antiretroviral therapy: A randomized controlled trial. Preventive Medicine Reports. 2021;21:101318.

100. El-Sadr WM, Beauchamp G, Hall HI, Torian LV, Zingman BS, Lum G, et al. Brief Report: Durability of the Effect of Financial Incentives on HIV Viral Load Suppression and Continuity in Care: HPTN 065 Study. Journal of Acquired Immune Deficiency Syndromes. 2019;81(3):300-3.

101. El-Sadr WM, Donnell D, Beauchamp G, Hall HI, Torian LV, Zingman B, et al. Financial Incentives for Linkage to Care and Viral Suppression Among HIV-Positive Patients: A Randomized Clinical Trial (HPTN 065). JAMA Internal Medicine. 2017;177(8):1083-92.

102. Farber S, Tate J, Frank C, Ardito D, Kozal M, Justice A, et al. A Study of Financial Incentives to Reduce Plasma HIV RNA Among Patients in Care. AIDS & Behavior. 2013;17(7):2293-300.

103. Gambone GF, Feldman MB, Thomas-Ferraioli AY, Shubert V, Ghose T. Integrating Financial Incentives for Viral Load Suppression into HIV Care Coordination Programs: Considerations for Development and Implementation. Journal of Public Health Management & Practice. 2020;26(5):471-80.

104. Haukoos JS, Witt MD, Coil CJ, Lewis RJ. The effect of financial incentives on adherence with outpatient human immunodeficiency virus testing referrals from the emergency department. Academic Emergency Medicine. 2005;12(7):617-21.

105. Lee J-Y, Lee JE, Moskowitz JT, Feaster DJ, Neilands TB, Dilworth SE, et al. An autoregressive cross-lagged model unraveling co-occurring stimulant use and HIV: Results from a randomized controlled trial. Drug & Alcohol Dependence. 2021;225:N.PAG-N.PAG.

106. Stitzer ML, Gukasyan N, Matheson T, Sorensen JL, Feaster DJ, Duan R, et al. Enhancing patient navigation with contingent financial incentives for substance use abatement in persons with HIV and substance use. Psychology of Addictive Behaviors. 2020;34(1):23-30.

107. Subramaniam S, Getty C-A, Holtyn AF, Rodewald A, Katz B, Jarvis BP, et al. Evaluation of a computer-based HIV education program for adults living with HIV. AIDS and Behavior. 2019;23(11):3152-64.

108. Adamson B, El-Sadr W, Dimitrov D, Gamble T, Beauchamp G, Carlson JJ, et al. The Cost-Effectiveness of Financial Incentives for Viral Suppression: HPTN 065 Study. Value in Health. 2019;22(2):194-202.

109. Alsan M, Beshears J, Armstrong WS, Choi JJ, Madrian BC, Nguyen MLT, et al. A commitment contract to achieve virologic suppression in poorly adherent patients with HIV/AIDS. AIDS. 2017;31(12):1765-9.

110. Anderson S, Jenner E, Lass K, Burgess S. Perspectives of HIV Clinic Staff on the Implementation of a Client Financial Incentives Program Targeting Viral Suppression. Journal of the Association of Nurses in AIDS Care. 2017;28(5):770-83.

111. Carrico AW, Neilands TB, Dilworth SE, Evans JL, Gomez W, Jain JP, et al. Randomized controlled trial of a positive affect intervention to reduce HIV viral load among sexual minority men who use methamphetamine. Journal of the International AIDS Society. 2019;22(12):e25436.

112. Gamble T, Branson B, Donnell D, Hall HI, King G, Cutler B, et al. Design of the HPTN 065 (TLC-Plus) study: A study to evaluate the feasibility of an enhanced test, link-to-care, plus treat approach for HIV prevention in the United States. Clinical Trials. 2017;14(4):322-32.

113. Ghose T, Shubert V, Poitevien V, Choudhuri S, Gross R. Effectiveness of a Viral Load Suppression Intervention for Highly Vulnerable People Living with HIV. AIDS & Behavior. 2019;23(9):2443-52.

114. Ghose T, Shubert V, Chaudhuri S, Poitevien V, Updyke A. Are Financial Incentives Appropriate Means of Encouraging Medication Adherence Among People Living With HIV? AMA Journal of Ethics. 2021;23(5):E394-401.

115. Greene E, Pack A, Stanton J, Shelus V, Tolley EE, Taylor J, et al. "It Makes You Feel Like Someone Cares" acceptability of a financial incentive intervention for HIV viral suppression in the HPTN 065 (TLC-Plus) study. PLoS ONE [Electronic Resource]. 2017;12(2):e0170686.

116. Holstad MM, DiIorio C, Magowe MK. Motivating HIV positive women to adhere to antiretroviral therapy and risk reduction behavior: the KHARMA Project. Online Journal of Issues in Nursing. 2006;11(1):5.

117. Javanbakht M, Prosser P, Grimes T, Weinstein M, Farthing C. Efficacy of an individualized adherence support program with contingent reinforcement among nonadherent HIV-positive patients: results from a randomized trial. Journal of the International Association of Physicians in AIDS Care: JIAPAC. 2006;5(4):143-50.

118. Jemison D, Jackson S, Oni O, Cats-Baril D, Thomas-Smith S, Batchelder A, et al. Pilot Randomized Controlled Trial of a Syndemics Intervention with HIV-Positive, Cocaine-Using Women. AIDS & Behavior. 2019;23(9):2467-76.

119. Maragh-Bass AC, Gamble T, Tolley EE. 'Either You Float or You Drown:' The Role of Social Ties and Stigma in Lived Experiences of the HIV Care Continuum in HPTN 065. AIDS & Behavior. 2020;24(9):2532-45.

120. Rigsby MO, Rosen MI, Beauvais JE, Cramer JA, Rainey PM, O'Malley SS, et al. Cue-dose training with monetary reinforcement: pilot study of an antiretroviral adherence intervention. Journal of General Internal Medicine. 2000;15(12):841-7.

121. Shelus V, Taylor J, Greene E, Stanton J, Pack A, Tolley EE, et al. It's all in the timing: Acceptability of a financial incentive intervention for linkage to HIV care in the HPTN 065 (TLC-Plus) study. PLoS ONE [Electronic Resource]. 2018;13(2):e0191638.

122. Silverman K, Holtyn AF, Rodewald AM, Siliciano RF, Jarvis BP, Subramaniam S, et al. Incentives for viral suppression in people living with HIV: A randomized clinical trial. AIDS and Behavior. 2019;23(9):2337-46.

123. Spratt ES, Papa CE, Mueller M, Patel S, Killeen T, Maher E, et al. Using Technology to Improve Adherence to HIV Medications in Transitional Age Youth: Research Reviewed, Methods Tried, Lessons Learned. Journal of General Medicine. 2017;1(1).

124. Tolley EE, Taylor J, Pack A, Greene E, Stanton J, Shelus V, et al. The Role of Financial Incentives Along the Antiretroviral Therapy Adherence Continuum: A Qualitative Sub-study of the HPTN 065 (TLC-Plus) Study. AIDS & Behavior. 2018;22(1):245-57.

125. Taylor NK, Buttenheim AM. Improving utilization of and retention in PMTCT services: can behavioral economics help? BMC Health Services Research. 2013;13:406.
